# Genetic Basis and Selection for Life-History Trait Plasticity on Alternative Host Plants for the Cereal Aphid *Sitobion avenae*

**DOI:** 10.1371/journal.pone.0106179

**Published:** 2014-09-02

**Authors:** Xinjia Dai, Suxia Gao, Deguang Liu

**Affiliations:** 1 State Key Laboratory of Crop Stress Biology for Arid Areas (Northwest A&F University), Yangling, Shaanxi Province, China; 2 Key Laboratory of Integrated Pest Management on Crops in Northwestern Loess Plateau, Ministry of Agriculture, Yangling, Shaanxi Province, China; 3 College of Plant Protection, Northwest A&F University, Yangling, Shaanxi Province, China; Oklahoma State, United States of America

## Abstract

*Sitobion avenae* (F.) can survive on various plants in the Poaceae, which may select for highly plastic genotypes. But phenotypic plasticity was often thought to be non-genetic, and of little evolutionary significance historically, and many problems related to adaptive plasticity, its genetic basis and natural selection for plasticity have not been well documented. To address these questions, clones of *S. avenae* were collected from three plants, and their phenotypic plasticity under alternative environments was evaluated. Our results demonstrated that nearly all tested life-history traits showed significant plastic changes for certain *S. avenae* clones with the total developmental time of nymphs and fecundity tending to have relatively higher plasticity for most clones. Overall, the level of plasticity for *S. avenae* clones’ life-history traits was unexpectedly low. The factor ‘clone’ alone explained 27.7–62.3% of the total variance for trait plasticities. The heritability of plasticity was shown to be significant in nearly all the cases. Many significant genetic correlations were found between trait plasticities with a majority of them being positive. Therefore, it is evident that life-history trait plasticity involved was genetically based. There was a high degree of variation in selection coefficients for life-history trait plasticity of different *S. avenae* clones. Phenotypic plasticity for barley clones, but not for oat or wheat clones, was frequently found to be under significant selection. The directional selection of alternative environments appeared to act to decrease the plasticity of *S. avenae* clones in most cases. G-matrix comparisons showed significant differences between *S. avenae* clones, as well as quite a few negative covariances (i.e., trade-offs) between trait plasticities. Genetic basis and evolutionary significance of life-history trait plasticity were discussed.

## Introduction

All organisms live in spatially and temporally variable and sometimes predictable environments. Organisms may cope with the highly variable environments by adaptation through genetic modifications under natural selection [1]. Phenotypic plasticity may also facilitate successful use of changing environments by an organism, and thus is considered to be another mechanism for adaptation [2]. However, the optimal strategy of organisms is assumed to be non-plastic and maximal in fitness traits for all environments, so one would rarely expect traits tightly linked to fitness to be plastic [3]. In addition, phenotypic plasticity was often considered to buffer the impact of natural selection, and thus act to slow evolutionary changes [4]. Therefore, phenotypic plasticity of an organism in response to variable environments had long been considered to be non-genetic and of little evolutionary importance until late 1980s [5]. Bradshaw coined the term ‘phenotypic plasticity’ to describe environmentally contingent morphological expression when developmental variability was considered to be uninteresting noise by most scientists [6]–[7]. But now it has been broadly used to describe all phenotypic responses to environmental changes [8]. Following Sultan [9], we define plasticity as variation in phenotypic expression of a genotype that occurs in response to particular environmental conditions. Plasticity of a particular trait may have positive (i.e., adaptive), negative (i.e., maladaptive) or no (i.e., neutral) consequences for a genotype’s fitness under different environments, so phenotypic plasticity can either retard or accelerate rates of phenotypic evolution based on relative fitness of the new phenotype [10]–[13]. Over the past two decades, interest in phenotypic plasticity has grown exponentially, and the change of interest reflects the new understanding that plasticity could be a powerful means of adaptation [7], [14]–[15]. Despite the large volume of work in this field, problems related to adaptive plasticity, genetic basis and natural selection for plasticity have not been well documented [14], [16]. All such problems are conceptually crucial for our understanding not only of evolutionary consequences of plasticity, but also of phenotypic evolution in general [16]–[17].

There exist fundamental differences in biological features among various organisms (e.g., plants and animals), which can have important implications for the evolution of phenotypic plasticity. For example, plants (sessile organisms) usually have greater plasticity than animals (mobile organisms) in morphological and developmental responses to changes in their biotic and abiotic environments, probably because animals can often move away from unfavorable environments [5], [18]. As a large and abundant group of organisms, http://en.wikipedia.org/wiki/Phenotypic_plasticity - cite_note-3#cite_note-3insect herbivores (many of them are highly mobile) have their own biological properties, and can often utilize a variety of host plant species. Significant variation in morphology, physiology and chemistry can occur among these plants [19]. Different host plants of insects often exist in temporally and spatially discrete patches that act as differential selective environments. Therefore, insect herbivores were also shown to be plastic in morphology, physiology, behavior or life-history in response to different host plants [4], [15], [20]–[21]. Surprisingly, studies on phenotypic plasticity of insects’ life-history traits on different host plants have been rare.

Aphids’ success in a wide diversity of ecosystems is partially attributed to their broad phenotypic plasticity in color, wing production and reproduction, although many of them are specialized on particular host plants [22]–[23]. The cotton aphid (*Aphis gossypii* Glover) or black bean aphid (*Aphis fabae*) was shown to be plastic in morphology [20], insecticide susceptibility [24], host choice behavior [21] or life-history traits (e.g., developmental times) [4]. Host plants (i.e., different environments) were found to be an important factor in inducing aphids’ plastic changes in phenotypes [4], [20]–[21]. Host plants also showed conditioning effects on the tested aphids, which is another piece of evidence for plasticity that happens without substantial modifications on the insect genome [21], [25]–[26]. Plasticity resulting from different host plants can play significant roles influencing the evolutionary trajectory of aphids. Surprisingly, studies on phenotypic plasticity of aphids’ life-history traits have been rare.

The cereal aphid, *Sitobion avenae* (Fabricius), can survive on numerous species in the Poaceae [27], and provides a good model to study life-history trait plasticity for insects that can disperse long distances. Several studies characterized key life-history traits (e.g., developmental times and fecundity) of *S. avenae* clones on wheat, barley and oat [25], [28]–[31]. In our previous study, we compared barley clones to oat clones from Shaanxi province, and found that barley and oat clones differentiated significantly in life-history traits, heritabilities of those traits, and the extent of specialization on a particular host plant; however, divergent selection on both host plants did not result in the formation of highly specialized clones or host races [23]. Therefore, differential adaptation of *S. avenae* clones to barley or oat in our previous study might result from their phenotypic plasticity. Since spatial heterogeneity and dispersal of organisms can have significant consequences for the evolution of plasticity [32], the study mentioned above had limitations in the number of host plants tested (i.e., two) and the source of tested *S. avenae* clones (from a single location) for analyzing the effects of plasticity. So, we collected *S. avenae* clones on three cereal crops from two provinces of China, and tested them in the laboratory. We hypothesize that key life-history traits of *S. avenae* was highly plastic in alternative environments, and the trait plasticity involved is genetically based and evolutionarily important. The aims of this study were to: 1) characterize phenotypic plasticity of key life-history traits for *S. avenae* clones in alternative environments (i.e., on alternative host plants); 2) assess the underlying genetic basis and natural selection for life-history trait plasticity of *S. avenae* clones; and 3) evaluate whether the observed plastic responses of *S. avenae* are adaptive.

## Materials and Methods

### Aphids and Plants

In order to increase genetic variability, *S. avenae* clonal genotypes were sampled from three host species and distinct locations from two provinces of China. Individual clones of *S. avenae* were collected in May of 2013 in Shaanxi Province, and in August of 2013 in Qinghai province. These clones came from barley, oat and wheat fields in the Shaanxi area (collected at three sites: 34°17′21.64″N, 108°4′10.09″E; 34°18′6.35″N, 108° 5′20.54″E; 34°18′35.73″N, 107°57′42.20″E) and Qinghai area (collected at three sites: 36°48′82.33″N, 101°59′89.94″E; 37°04′87.06″N, 101°90′00.31″E; 37°13′04.89″N, 101°28′78.21″E) (no specific permissions were required for the sample collecting activities at all the abovementioned sites, and no endangered or protected species were involved in the collecting activities). In order to limit the chance of re-sampling individuals from the same parthenogenetic mother, an individual wingless adult aphid was collected from a plant separated by at least 10 m from other samples [25]. At least 20 different clones were collected for each plant species in each area, and they were used to start separate colonies in the laboratory. Aphid clones were cultured on the species of plants from which they were originally collected (i.e., barley, oat or wheat). Wheat (*Triticum aestivum* L. cv. Aikang 58), oat (*Avena sativa* L. cv. Sandle) and barley (*Hordeum vulgare* L. cv. Xian 91–2) seeds were planted in plastic pots (6 cm in diameter) containing turfy soil, vermiculite and perlite (4∶3:1, v/v/v). Plants with collected clones were enclosed with a transparent cylinder (5.5 cm in diameter, 15 cm in height) which had a Terylene mesh top for ventilation, and maintained in rearing rooms (at 20±2°C and a photoperiod of 16∶ 8 (L: D)). Plants were watered or replenished as needed. Prior to the bioassays, aphid clones were reared for two generations under common conditions in the lab to minimize the effects of confounding factors (e.g., weather conditions) according to Pitchers et al. [33]. After that, aphid clones were randomly selected from the colony for use in the following tests.

### Life History Data Collection

Barley, oat and wheat seedlings (one per pot) used in life-history tests were planted as described above. When they reached one- to two-leaf stage, seedlings then received aphids that were transferred from rearing plants. To have a cohort of first instar nymphs with the same age, young wingless female adults of 16 different clones (10 from Shaanxi and 6 from Qinghai) for each plant species were transferred to test plants (one individual per plant). After 2–3 h, plants were checked and all aphids except one newborn nymph in each pot were removed by a fine paint brush. To prevent the aphids from escaping, each pot of plant was enclosed with a transparent plastic cylinder described above. Test plants were maintained in environmental growth chambers at 20±1°C, a relative humidity of 65±2%, and a photoperiod of 16∶8 (L:D). Four to six replicates were established on each test plant species for each clone. All aphid clones were tested on both original and alternate host species (i.e., barley, oat and barley). Each test aphid individual was observed twice daily from birth until the onset of reproduction, and molting and mortality were recorded at about the same time each day. After the initiation of reproduction, which usually occurred 1 d after the fourth molt, mortality and fecundity of aphids were recorded daily, and their offspring were then removed from each test plant daily for 7 d.

### Statistical Analysis

Developmental times of first to fourth nymphal instars (hereafter referred to as DT1 to DT4), the total developmental time of nymphs (from birth to adult emergence) (hereafter referred to as DT5), and 7-d fecundity (nymphs born in the first 7 d after the initiation of reproduction) were calculated. The abovementioned fitness traits were analyzed with three way nested analysis of variance (ANOVA), which was conducted with clones nested in plant origin (i.e., barley, oat and wheat) in SAS [34]. The main effects of location, plant origin and test plant were analyzed, and the interaction between the latter two was also considered. When the overall variation in ANOVA was significant, post-hoc comparisons among means were carried out by using Tukey tests at α = 0.05.

The amount of plasticity was evaluated by calculating the coefficient of variation (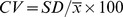; *SD*, standard deviation of treatments; 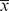, mean of treatments) for each trait in different environments (i.e., on different hosts). Another nested ANOVA with abovementioned factors was performed to analyze the phenotypic plasticity of life-history traits and genetic variation underlying phenotypic plasticity. Data were log-transformed if needed to meet the assumptions of normality and homoscedasticity required for these analyses.

Our experimental design with clonal genotypes allows us to estimate the total variance of a particular trait (*V_P_*), which includes among-clone genetic components *V* ***_G_***, the (broad sense) genetic variance, and within-clone components *V_E_* (i.e., environmental variance or residual variance). Broad-sense heritabilities were calculated as the proportion of the total variance accounted for by the among-clone variance component (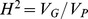). The statistical significance of heritabilities was assessed by using likelihood-ratio tests (LRTs) following Carter et al. [35].

Genetic variance and covariance estimates for life-history trait plasticities were obtained with the restricted maximum likelihood (REML) method implemented in the software VCE 6.0.2 [36]. The genetic correlation between traits *x* and *y* was calculated from the genetic covariance estimate (cov[*x, y*]) and their additive variances as 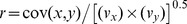. The resulting G matrices were compared using the Flury hierarchical method, using the software CPCrand [37]. Based on maximum likelihood, this method can analyze structural differences among G matrices by comparing their eigenvectors and eigenvalues. Specifically, the method can test the following models in order: (1) unrelated structure (meaning that matrices do not share any eigenvector), (2) partial common principal component (matrices sharing some eigenvectors), (3) common principal components (matrices sharing all eigenvectors but not eigenvalues), (4) proportionality (matrices with same eigenvectors and proportional eigenvalues), and (5) equality (matrices with same eigenvectors and eigenvalues) (see also in [35]). The significance of genetic covariances and correlations between trait plasticities were evaluated using LRTs following Carter et al. [35].

In this study, we used 7 d fecundity as the fitness estimate [23], [31]. Relative fitness of an aphid clone was calculated by dividing the clone’s 7 d fecundity by the mean of all clones in each treatment. All traits were standardized to mean zero and unit variance. We then calculated univariate standardized selection differentials using parametric regression analysis following Lande and Arnold [38] to quantify the strength of selection for *S. avenae* on the three cereals. Selection differentials can estimate the total strength of selection on a trait, and thus include both direct selection and indirect selection arising through covariances with other traits [39]. To separate the effects of direct selection on focal traits from the effects of indirect selection on other traits, standard linear selection gradients were estimated by performing multiple regressions following Lande and Arnold [38]. Regression analyses were performed using the PROC REG procedure in SAS [34].

Principal component analysis (PCA) (Proc PRINCOMP) was performed with plasticities of all life-history traits measured above [34]. The factor weightings for each replicate in the PCA mentioned above were calculated and the resulting values were used as a composite plasticity factor in subsequent regression analyses. The PROC REG procedure in SAS [34] was used to identify the relationships between the relative fitness of *S. avenae* clones on test plants and the level of life-history plasticity (i.e., the composite plasticity factor calculated as the first component extracted from PCA).

## Results

### Life-history trait plasticity

**Figure 1:**
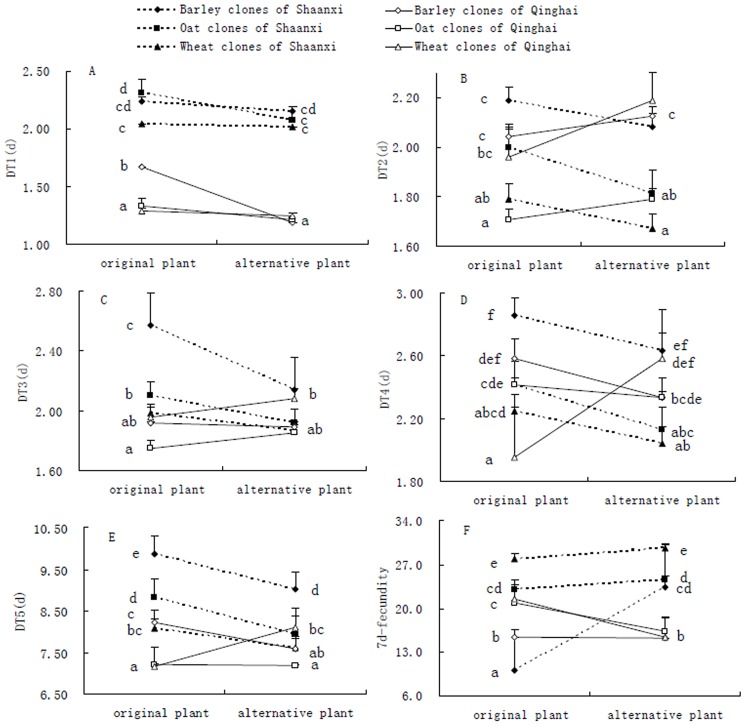
Comparisons of life-history traits for barley, oat and wheat clones of *Sitobion avenae* from two areas on original and alternative host plants, showing mean plasticity of tested clones (A-D for DT1-DT4, the developmental time of 1^st^ to 4^th^ instar nymphs; E for DT5, the total developmental time of nymphs; F for 7-d fecundity; data for a particular trait with different letters were significantly different at the *P*<0.05 level, ANOVA followed by Tukey tests).

After transfer to alternative environments (i.e., alternative host plants), all tested clones from both areas showed non-significant changes in DT1 except the barley clones from the Qinghai area (Fig. 1A). DT1 of barley clones from Qinghai was significantly reduced in alternative environments, showing higher mean plasticity of these clones in comparison to other clones (Fig. 1A). DT2 presented non-significant changes for all clones (i.e., barley, oat and wheat) from both areas, indicating similar levels of mean plasticity for them (Fig. 1B). Barley clones from the Shaanxi area showed significantly lower DT3 in alternative environments (meaning relatively higher mean plasticity), but all other clones presented relatively lower mean plasticity in DT3, indicated by non-significant changes in DT3 in different environments (Fig. 1C). After they were transferred from plants of origin to alternative ones, significant changes in DT4 were found for oat clones from Shaanxi and wheat clones from Qinghai; non-significant changes were found for all other clones tested (Fig. 1D). After switching environments, all clones presented significant changes in DT5 but oat clones from Qinghai, indicating that DT5’s mean plasticity was relatively higher for *S. avenae* clones in comparison to other trait plasticities (Fig. 1E). Significant changes in 7-d fecundity were identified for all clones of both areas but wheat clones of Shaanxi and barley clones from Qinghai, showing relatively higher mean plasticity of 7-d fecundity for the majority of *S. avenae* clones (Fig. 1F).

### Genetic variation of trait plasticity

The plasticity for the developmental time of 1^st^ instar nymphs was significantly influenced by ‘location’, ‘origin’, and interaction between ‘origin’ and ‘test’, as well as ‘clone’ (nested in plant origin) (Table 1). All factors (i.e., ‘location’, ‘origin’, ‘test’, ‘origin x test’, and ‘clone’ nested in plant origin) showed significant effects on the plasticity for the developmental times of 2^nd^ to 4^th^ instar nymphs. Similar results were found for the plasticities for the total developmental time of nymphs and 7-d fecundity. The significant interactions between ‘plant origin’ and ‘test plant’ for the plasticity of all tested life-history traits indicated differences in host adaptation among different clones. The factor ‘clone’ alone accounted for a significant proportion (i.e., 27.7–62.3%) of the total variance for the plasticity of life-history traits mentioned above. ‘Clone’ and ‘origin’ together explained 48.6–68.6% of the total variance, and ‘location’ accounted for 1.3–19.3%, whereas ‘test’ and ‘origin x test’ contributed relatively little (i.e., 0.7–3.7% and 1.1–6.5%, respectively) to the total variance. So, genetic effects were evident for the plasticity of all the fitness traits tested.

**Table 1:** Estimates of variance components for trait plasticities of *Sitobion avenae* clones showing main effects of collecting locations (location), plant origin (origin), test plant (test), clone nested in origin and interactions (significant effects highlighted in boldface).

Trait	Variance source	df	*F*	*P*	% total
Developmental time of 1^st^ instar nymphs	Location	1	10.74	**0.001**	**1.3**
	Origin	2	24.20	**<0.001**	**6.0**
	Test	2	2.79	0.063	0.7
	Origin×test	1	27.98	**<0.001**	**3.5**
	Clone(origin)	38	10.98	**<0.001**	**52.1**
	Error	291	−	−	36.3
Developmental time of 2^nd^ instar nymphs	Location	1	92.47	**<0.001**	**10.6**
	Origin	2	3.52	**0.03**	**0.8**
	Test	2	3.76	**0.02**	**0.9**
	Origin×test	1	56.59	**<0.001**	**6.5**
	Clone(origin)	38	10.97	**<0.001**	**47.8**
	Error	291	−	−	33.4
Developmental time of 3^rd^ instar nymphs	Location	1	68.40	**<0.001**	**6.2**
	Origin	2	20.91	**<0.001**	**3.8**
	Test	2	17.30	**<0.001**	**3.1**
	Origin×test	1	16.33	**<0.001**	**1.5**
	Clone(origin)	38	17.10	**<0.001**	**59.0**
	Error	291	−	−	26.4
Developmental time of 4^th^ instar nymphs	Location	1	72.26	**<0.001**	**5.8**
	Origin	2	39.82	**<0.001**	**6.3**
	Test	2	8.24	**<0.001**	**1.3**
	Origin×test	1	14.36	**<0.001**	**1.1**
	Clone(origin)	38	20.56	**<0.001**	**62.3**
	Error	291	−	−	23.2
Total Developmental time of nymphs	Location	1	99.72	**<0.001**	**10.3**
	Origin	2	42.58	**<0.001**	**8.8**
	Test	2	17.64	**<0.001**	**3.7**
	Origin×test	1	62.29	**<0.001**	**6.5**
	Clone(origin)	38	10.31	**<0.001**	**40.6**
	Error	291	−	−	30.1
7-d fecundity	Location	1	472.05	**<0.001**	**19.3**
	Origin	2	442.41	**<0.001**	**36.2**
	Test	2	43.62	**<0.001**	**3.6**
	Origin×test	1	30.64	**<0.001**	**1.3**
	Clone(origin)	38	17.81	**<0.001**	**27.7**
	Error	291	−	−	11.9

*Sitobion avenae* clones from barley, oat and wheat presented little differentiation in broad-sense heritability of life-history trait plastcities (Table 2). The broad-sense heritabilities for all trait plasticities of all clones were high and significant except for DT1 of barley clones from both areas. Heritabilities were particularly high for the plasticities of DT3, DT4, DT5 and 7-d fecundity for barley clones from Shaanxi, and for those of DT5 for barley clones from Qinghai and 7-d fecundity for oat or wheat clones from Qinghai.

**Table 2:** Broad-sense heritabilities (SE) of life history trait plasticities for different *Sitobion avenae* clones from barley, oat and wheat on alternative host plants.

Traits	Clone sources					
	Shaanxi area			Qinghai area		
	Barley	Oat	Wheat	Barley	Oat	Wheat
DT1	0.0402n (0.102)	0.6398*** (0.121)	0.6566*** (0.102)	0.3123n (0.201)	0.6529* (0.187)	0.4998* (0.203)
DT2	0.5908* (0.221)	0.5286** (0.194)	0.5291*** (0.053)	0.6117* (0.197)	0.4333** (0.167)	0.4932*** (0.083)
DT3	0.8759*** (0.053)	0.5273*** (0.063)	0.4037** (0.179)	0.6817*** (0.128)	0.3852** (0.122)	0.3265* (0.167)
DT4	0.8907*** (0.033)	0.7834*** (0.086)	0.7852*** (0.085)	0.5077** (0.107)	0.7726** (0.173)	0.6165*** (0.093)
DT5	0.8088*** (0.082)	0.7628*** (0.095)	0.7886*** (0.080)	0.8156*** (0.065)	0.7315*** (0.096)	0.7955*** (0.105)
7 d fecundity	0.9432*** (0.039)	0.7016*** (0.105)	0.7651*** (0.088)	0.7469*** (0.088)	0.8026** (0.120)	0.8251*** (0.073)

Note: DT1-DT4, the developmental time of 1^st^ to 4^th^ instar nymphs; DT5, the total developmental time of nymphs; statistical significance of heritability for a trait within clones (i.e. barley, oat, wheat) evaluated using likelihood-ratio tests; *, *P*<0.05; **, *P*<0.01; ***, *P*<0.001.

Significant genetic correlations were found between plasticities of life-history traits for all clones from both areas (Table 3). All trait-plasticity pairs for barley clones from the Qinghai area presented significant genetic correlations but DT1-DT3, DT2-DT3, DT2-DT5, and DT2-7d fecundity, and all significant correlations for these clones were positive except the pair of DT1-DT2. For barley clones from Shaanxi, Plasticity of DT1was correlated with none of all other trait plasticities, which were significantly correlated with one another; the only significantly negative correlation (i.e., trade-off) for these clones was found between plasticities of DT2 and 7-d fecundity. Similar patterns were found for oat and wheat clones from both Qinghai and Shaanxi, where the majority of trait-plasticity pairs showed significant correlations, and all significant correlations were positive with few exceptions. The only negative correlations of oat clones were found for the pairs of DT2-DT4 and DT2-DT5 of clones from Qinghai, and for DT1-DT3 and DT1-7 d fecundity from Shaanxi. The only significantly negative correlation of wheat clones was between plasticities of DT3 and 7 d fecundity for clones from Qinghai.

**Table 3:** Genetic correlations among life history trait plasticities for different *Sitobion avenae* clones from barley, oat and wheat in two areas.

Trait-plasticity pairs	Clone sources					
	The Qinghai area			The Shaanxi area		
	Barley	Oat	Wheat	Barley	Oat	Wheat
DT1-DT2	−0.9079**	0.0492	0.3974*	−0.0298	0.2332*	0.3634*
DT1-DT3	0.0480	0.5457**	−0.0545	0.0658	−0.2651*	0.1720*
DT1-DT4	0.5144**	0.0407	0.2627*	0.0308	0.0602	0.2832*
DT1-DT5	0.3264*	0.3767*	0.1778*	0.0958	0.3304*	0.1812*
DT1-7 d fecundity	0.3816*	0.3511*	0.0926	−0.0489	−0.2455*	−0.0846
DT2-DT3	0.1221	−0.1156	−0.1479	0.1964*	−0.0943	0.0999
DT2-DT4	0.3761*	−0.1558*	0.3252*	0.1614*	−0.0985	0.3825**
DT2-DT5	−0.1324	−0.1924*	0.2670*	0.2843*	0.3874**	−0.0046
DT2-7 d fecundity	−0.1279	0.0425	0.1822*	−0.3327*	−0.0464	0.1560*
DT3-DT4	0.2112*	0.1041	0.1560	0.8745***	0.2259*	0.3118*
DT3-DT5	0.6200**	0.2999*	0.0703	0.8429***	0.1953*	0.3133*
DT3- 7 d fecundity	0.2548*	0.2546*	−0.3055*	0.2577*	0.3048*	0.0046
DT4-DT5	0.3724**	0.4431**	0.4312**	0.8271***	0.5573**	0.5580**
DT4-7 d fecundity	0.4789**	0.4762**	0.1668*	0.4057**	0.2568*	0.5298**
DT5-7 d fecundity	0.4740**	0.4965**	0.5531**	0.4536**	0.2848*	0.1787*

Note: DT1-DT4, the developmental time of 1^st^ to 4^th^ instar nymphs; DT5, the total developmental time of nymphs; statistical significance of genetic correlations evaluated using likelihood-ratio tests; *, *P*<0.05; **, *P*<0.01; ***, *P*<0.001.

G-matrix comparisons by Flury’s method and jump-up approach (that is, at each step in the hierarchy, the hypothesis is tested against the hypothesis of unrelated structure) showed significant differences between paired matrices of life-history trait plasticities (Table 4). The difference between G matrices for barley and oat clones from both areas was best explained by the full CPC model (i.e., all principal components shared in common), but the matrices were not equal (for Shaanxi: LRT = 90.5, *P*<0.001; for Qinghai: LRT = 332.6, *P*<0.001). The CPC(4) model best explained the differences between matrices for barley and wheat clones (LRT = 128.1, *P*<0.001), and between those for oat and wheat clones (LRT = 47.7, *P*<0.001) from the Shaanxi area, in other words, matrices shared four of the six possible principal components. However, unrelated structures were found between matrices for barley and wheat clones (LRT = 362.7, *P*<0.001), and between those for oat and wheat clones (LRT = 52.6, *P*<0.001) from the Qinghai area.

**Table 4:** Comparisons of G-matrices for life-history trait plasticities of barley, oat and wheat clones of *Sitobion avenae* in two areas.

Clone source	G matrices	Flury hierarchy		
		LRT	*P*-value	Verdict
The Shaanxi area	Barley vs. oat	90.5	<0.001	Full CPC
	Barley vs. wheat	128.1	<0.001	CPC(4)
	Oat vs. wheat	47.7	<0.001	CPC(4)
The Qinghai area	Barley vs. oat	332.6	<0.001	CPC
	Barley vs. wheat	362.7	<0.001	Unrelated
	Oat vs. wheat	52.6	<0.001	Unrelated

Note: The verdict of the Flury hierarchy is the model shown to be the best in explaining the difference between paired matrices; the *P*-values are for the test of equality of two matrices; full CPC, all principal components shared in common; CPC(4), four of the six possible components shared in common; unrelated, no relations between the matrices.

### Selection of alternative environments on trait plasticity

Directional selection differentials and gradients were estimated for barley, oat and wheat clones from both areas (Table 5). Barley clones from the Qinghai area presented significantly negative differentials for the plasticity of all life-history traits tested but DT2 and DT3. For barley clones from Qinghai, the directional selection gradients of plasticity for DT1 and DT4 were significantly negative, those for DT2 and DT3 significantly positive, and those for DT5 and 7-d fecundity non-significant. All selection coefficients for barley clones from the Shaanxi area were significant except the selection gradient of plasticity for DT2 and selection differentials of plasticity for DT1 and DT2, and all the significant selection coefficients were negative except the selection gradient of plasticity for DT5. Oat clones from Qinghai showed significantly negative selection coefficients (i.e., differential and gradient) for plasticity of DT4 and 7-d fecundity, the selection differential of DT5 plasticity was also significantly negative, and all other selection coefficients for these clones were non-significant. The only significant coefficient for oat clones from Shaanxi was the selection gradient of DT2 plasticity. Wheat clones from Qinghai had a significantly negative differential and gradient for DT5 plasticity, and they also had a significantly negative selection differential for DT2 plasticity. Alternative host plants also showed little selection for life-history trait plasticity of wheat clones from Shaanxi, and the only significant selection coefficients were the selection differential of plasticity for DT4, and the selection differential and gradient of plasticity for 7-d fecundity.

**Table 5:** Selection differentials and gradients for life-history trait plasticities of *Sitobion avenae* clones collected from barley, oat and wheat in two areas.

Traits	Barley clones		Oat clones		Wheat clones	
	Differential	Gradient	Differential	Gradient	Differential	Gradient
**For clones from the Qinghai area**						
DT1	−0.1425***	−0.0659*	−0.0655	−0.0090	0.0048	0.0631
DT2	0.0495	0.0763*	−0.0288	−0.0286	−0.0860*	−0.0775
DT3	0.0091	0.0815*	−0.0328	0.0294	0.0024	0.0350
DT4	−0.1368***	−0.1045**	−0.1991***	−0.1161***	−0.0568	0.0366
DT5	−0.1104**	−0.0743	−0.1259**	0.0212	−0.1584***	−0.1999***
7-d fecundity	−0.1341***	−0.0333	−0.2480***	−0.2057***	−0.0916*	0.0487
**For clones from the Shaanxi area**						
DT1	−0.0342	−0.0384**	−0.0201	−0.0215	−0.0264	−0.0257
DT2	0.0262	−0.0042	0.0158	0.0290*	−0.0260	−0.0026
DT3	−0.0881***	−0.0895**	0.0174	0.0208	0.0075	0.0220
DT4	−0.0986***	−0.0597*	−0.0030	0.0094	−0.0499***	−0.0245
DT5	−0.0784***	0.0959**	−0.0063	−0.0149	−0.0219	−0.0027
7-d fecundity	−0.0927***	−0.0919***	−0.0090	−0.0176	−0.0531***	−0.0413*

Note: DT1-DT4, the developmental time of 1^st^ to 4^th^ instar nymphs; DT5, the total developmental time of nymphs; *, *P*<0.05; **, *P*<0.01; ***, *P*<0.001.

### Relationship between plasticity and fitness

**Figure 2:**
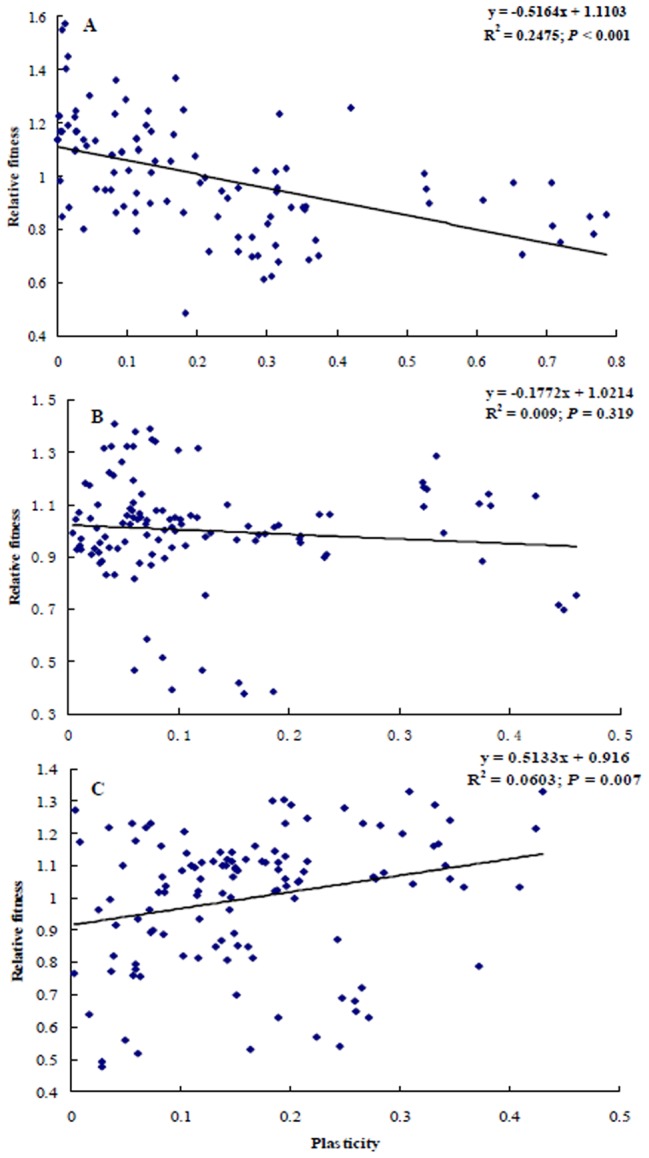
Relationship between total life-history trait plasticity and relative fitness of *Sitobion avenae* clones collected from barley (A), oat (B) and wheat (C) (the amount of plasticity evaluated by calculating the coefficient of variation for each trait in different environments; total life-history trait plasticity calculated from the first factor extracted from the principal component analysis of all life-history trait plasticities).

The relative fitness of *S. avenae* clones was regressed against the first factor (PC1) extracted from PCA of all life-history trait plasticities. The results of PCA for all clones showed the first three components explaining 83.2% (45.9% for PC1) of the total data variability. Barley clones with higher plasticity tended to have lower fitness, whereas the fitness of wheat clones tended to rise with increasing plasticity (Fig. 2). The linear relationship between relative fitness and plasticity was found to be significant for barley clones (Fig. 2A, R ^2^ = 0.25, *P*<0.001) or wheat clones (Fig. 2C, R ^2^ = 0.06, *P*<0.01). But the linear relationship between fitness and plasticity was not significant for oat clones (Fig. 2B).

## Discussion

### Genetic basis of phenotypic plasticity

Phenotypic plasticity is considered a universal quality of life, but it’s often neglected and thought to be non-genetic historically (until late 1980s) [4]–[7], [40]. Therefore, studies on the genetic basis of phenotypic plasticity in insects have been rare, especially for their key life-history traits. Our tests of *S. avenae* clones on barley, oat and wheat showed that the factor ‘clone’ accounted for a significant proportion (i.e., 27.7–62.3%) of the total variance for the life-history trait plasticities, indicating that the divergence of plasticity among *S. avenae* clones had a genetic basis. Another piece of evidence for the genetic basis of plasticity is that nearly all plasticities of life-history traits for all tested clones showed significant heritability with very few exceptions. Significant genetic correlations were also found between plasticities of life-history traits for all tested *S. avenae* clones, which provided an additional piece of information regarding the genetic basis of phenotypic plasticity. Three genetic models have been proposed to explain phenotypic plasticity, and they are overdominance, pleiotropy, and epistasis [3]. The overdominance model states that plasticity decreases with increasing heterozygosity (i.e., the more heterozygous a clone, the less plastic it will be), and this model can be important for explaining plasticity in aphids that frequently show high heterozygote excess due to parthenogenesis [41]–[42]. The genetic mechanisms that underlie plastic response are still poorly understood [43]. Further studies with tested clones using microsatellites can clarify the relationship between heterozygosity and plasticity in aphids, and improve our understanding of genetic basis for plasticity in aphids.

### Selection on life-history trait plasticity

Alternative environments can impose natural selection not only on life-history traits, but on their plasticity. Questions related to how (and how frequently) natural selection acts on plasticity are conceptually crucial for our understanding not only of genotype-by-environment interactions, but also of phenotypic evolution in general [16]. In our study, there was substantial selection directly on the plasticity of developmental times for barley clones, which was indicated by their significant selection gradients. However, there was little such selection for oat or wheat clones. Substantial direct selection on the plasticity of fecundity was also found for certain clones of barley, oat and wheat. The direct selection of alternative environments acted to decrease the plasticity of *S. avenae*’s life-history traits in most cases except that it increased the plasticity of developmental times of certain nymphal instars for barley clones. Alternative environments produced positive direct selection on the plasticity of total developmental time of nymphs for barley clones from Shaanxi area, but this was masked in the selection differential by indirect selection through some correlated characters. However, differing signs on the coefficients for the directional selection gradient of the developmental times for barley clones were found, so selection could act separately on the trait plasticities involved. Therefore, our study revealed significant directional selections on the developmental times and fecundity of *S. avenae*, but the identified directional selection acted to decrease the plasticity in most cases. The results appeared to be in agreement with the findings that some *S. avenae* clones were specialized to a certain extent on different hosts [23], [25], [44], since highly plastic genotypes may lower their plasticity to become relatively specialized to certain environment. Other relatively generalized clones may be sufficiently plastic to survive well on the three cereals (i.e., barley, oat and wheat), so that natural selection will not occur to produce specialized ecotypes. Although phenotypic plasticity is a common phenomenon in aphids, it appears that highly plastic clones of *S. avenae* have been removed from the population by natural selection. It is assumed that traits that are tightly linked to fitness should be more strongly canalized as a result of past stabilizing selection [3], [45]. Our finding is consistent with the abovementioned assumption, since developmental times and fecundity of an organism are both key fitness components.

### Evolutionary significance of plasticity

After cereal crops are harvested in the summer, some individuals of *S. avenae* may disperse a short distance to find wild grass hosts, others may have to move long distance northward to find other cereal crop fields. So, a *S. avenae* clone may experience several host plant species in a single year. This pattern might lead to the maintenance of moderate phenotypic plasticity in response to changes in host plant species. It makes sense to assume that a clone with higher plasticity can become established more easily in alternative environments than that with low plasticity. Adaptive phenotypic plasticity can evolve in natural populations, which is suggested by the frequent observation of genetic variation for plasticity, but direct experimental evidence is rare due to logistical reasons [16]. In our study, some wheat clones of *S. avenae* with higher level of life-history trait plasticity tended to have higher fitness, indicating that phenotypic plasticity can be adaptive for these clones. Positively affected clones may reinforce the relationship between plasticity and fitness during feeding on alternative host plants. Significant heritability and genetic correlations for *S. avenae*’s life-history trait plasticities identified in our study also indicated the evolutionary potential of adaptive plasticity. However, evidence for adaptive plasticity was not found for oat or barley clones. Cost of plasticity was an important factor influencing evolution of adaptive plasticity for insect populations experiencing heterogeneous environments [46]. So, the cost of being plastic could be high for barley or oat clones. Another mutually non-exclusive explanation is that a population that inhabits heterogeneous environments may be selected to evolve a genetic constitution that allows different levels of phenotypic plasticity to adjust to different environments so as to increase its overall fitness [47].

The evolution of adaptive plasticity can also be influenced by the structure of G-matrix for *S. avenae*’s life-history trait plasticities. Interestingly, quite a few negative covariances (i.e., trade-offs) were found between trait plasticities. These trade-offs (also shown by negative genetic correlations) may also playing a role in slowing the evolution of highly plastic *S. avenae* clones. Significant differences between matrices for barley, oat and wheat clones were found, which have important implications for the overall direction of plasticity evolution in *S. avenae*. The G-matrix structure of different traits for aphid clones have complex relationships with factors like clone specialization, trade-offs, and genotype-by-environment interactions [48]. It may be interesting to determine the stability of the G-matrix for life-history trait plasticity of *S. avenae* over time and further explore its evolutionary implications in future studies.

The selection of clones with relatively low life-history trait plasticity was found for barley clones in our study. This may facilitate the process where *S. avenae* clones become specialized to a certain host plant. Recently, plasticity has been widely recognized as a significant mode of phenotypic diversity and hence as an important aspect of how organisms evolve in different environments [43]. It has been pointed out that speciation in herbivore species can start with phenotypic plasticity, not necessarily with reproductive isolation, therefore, the presence of sufficient variation in phenotypic plasticity of aphids may facilitate host race formation and sympatric speciation [4]. A recent study showed that five unique *S. avenae* biotypes might have developed on commonly planted wheat varieties in China [49]. It appears that perfect plasticity is hard to evolve for *S. avenae*, but plasticity may be important for the evolution of specialized genotypes. This leads to the challenging question of whether phenotypic plasticity is the raw material for speciation and biodiversity [45]. Further studies with costs of life-history trait plasticity may provide insights into its evolution, as well as its significance in host race formation and sympatric speciation for *S. avenae*.
